# [Retracted] Phenotypic characterization of human prostatic stromal cells in primary cultures derived from human tissue samples

**DOI:** 10.3892/ijo.2026.5916

**Published:** 2026-07-13

**Authors:** Giovanni Luca Gravina, Andrea Mancini, Guido Ranieri, Boris Di Pasquale, Francesco Marampon, Luigi Di Clemente, Enrico Ricevuto, Claudio Festuccia

Int J Oncol 42: 2116-2122, 2013; DOI: 10.3892/ijo.2013.1892

Following the publication of the above paper, it was drawn to the Editor's attention by a concerned reader that, regarding Figs. 2A and 4B (and possibly also Fig. 4A), certain of the gel lanes within these figure parts (showing numbered cultures) appeared to contain duplicated data (specifically, lanes 2-4 and 8-10 in Fig. 2A, lanes 5 and 6 in Fig. 4A, and lanes 1-4 and 7-10 in Fig. 4B). Upon performing an independent analysis of the data in this paper, it also came to light that the β-actin data in Fig. 3A and the Desmin data in Fig. 3E had appeared in a previous paper published in *American Journal of Pathology* that featured entirely different authors, although comparing the affiliations of the papers, one of the universities was the same.

Given the apparent duplications of data within this paper itself and the re-use of data that had originally appeared in a different article, the Editor of *International Journal of Oncology* has decided that it should be retracted from the Journal. The authors were asked for an explanation to account for these concerns, but the Editorial Office did not receive a satisfactory reply. The corresponding author, Claudio Festuccia, takes full responsibility for the raised concerns and clarifies that the other co-authors were not directly involved in the preparation of the submitted figures. The Editor apologizes to the readership for any inconvenience caused.

